# UV-irradiating synthesis of cyclodextrin–silver nanocluster decorated TiO_2_ nanoparticles for photocatalytic enhanced anticancer effect on HeLa cancer cells

**DOI:** 10.3389/fchem.2022.995261

**Published:** 2022-09-27

**Authors:** Hongying Wang, Ze Xing, Yan Sun, Yingjie Jing, Jian Zhang, Xinyao Li, Hailiang Zhang, Adnan Shakoor, Junsheng Guo

**Affiliations:** ^1^ Thoracic Trauma and Oncology Institute, Chifeng University, Chifeng, China; ^2^ Department of Respiratory and Critical Care Medicine, Chifeng University Affiliated Hospital, Chifeng, China; ^3^ Department of Respiratory and Critical Care Medicine, The Affiliated Nanhua Hospital, Hengyang Medical School, University of South China, Hengyang, China; ^4^ Department of Oncology Medicine, Inner Mongolia Medical University Affiliated Hospital, Hohhot, China; ^5^ Department of Urology, Chifeng University Affiliated Hospital, Chifeng, China; ^6^ School of Stomatology, Chifeng University, Chifeng, China; ^7^ Guangdong Huace Biomedical Research Center, Guangzhou, China; ^8^ Department of Control and Instrumentation Engineering, King Fahd University of Petroleum and Minerals, Dhahran, Saudi Arabia

**Keywords:** TiO_2_, cyclodextrin-silver, photocatalytic, anticancer, HeLa, cervical

## Abstract

Titanium dioxide (TiO_2_) has emerged as a viable choice for several biological and environmental applications because of its high efficiency, cheap cost, and high photostability. In pursuit of this purpose, the research of its many forms has been influenced by these unique aspects. The development of novel TiO_2_-based hybrid materials with enhanced photocatalytically induced anticancer activity has gained tremendous attention. Here, we have developed a novel photocatalytic material (TiO_2_–Ag NPs@-CD) by decorating ultrasmall silver nanoparticles (Ag NPs) with per-6-thio-β-cyclodextrin (SH-β-CD) on TiO_2_ NPs. TiO_2_–Ag NPs@-CD were characterized by employing various characterization techniques and evaluated for their anticancer activity against HeLa cancer cells using an MTT assay. The biocompatibility of the designed nanoparticles was determined on two normal cell lines, namely, 3T3 and human mesenchymal stem cells (hMSCs). The results show that the TiO_2_–Ag NPs@-CD induced superior cytotoxic effects on HeLa cancer cells at a concentration of 64 μg/ml. Live-dead staining and oxidative stress investigations demonstrated that cell membrane disintegration and ROS-induced oxidative stress generated by TiO_2_-Ag NPs@-CD inside HeLa cancer cells are the contributing factors to their exceptional anti-cancer performance. Moreover, TiO_2_-Ag NPs@-CD exhibited good biocompatibility with 3T3 and hMSCs. These results indicated that the combination of all three components—a silver core, SH-β-CD ligands, and TiO_2_ nanoparticles—produced a synergistic anticancer effect. Hence, the TiO_2_-Ag NPs@-CD is a promising material that can be employed for different biological applications.

## Introduction

Over the past few decades, cancer has become the second-leading cause of death globally (Siegel et al., 2020). In terms of morbidity and prevalence, cervical cancer (CCA) is a disease that affects women and is ranked fourth globally (Canfell et al., 2020). Although human papillomavirus vaccination, new chemotherapeutic drugs, and other therapeutic techniques provide efficient control and treatment of cervical cancer, metastatic CCA is an acute or chronic cancer that urgently requires novel anticancer drugs and therapeutic strategies for treatment (Brisson et al., 2020; Cohen et al., 2020).

As nanotechnology has advanced, nanomaterials have been considered for anticancer applications (Khan et al., 2018a, 2018b, 2021; Khan and Lee, 2020; Lu et al., 2021). Numerous studies have been conducted on photocatalysts that use distinct portions of the solar spectrum. The electrons and hole pairs, created by the absorption of visible light on photocatalytic nanomaterials, can independently react with water and oxygen. Once reacted with oxygen and water, they make a variety of reactive oxygen species (ROS) (Yang et al., 2022). The cell membrane may become oxidatively damaged as a result of these ROS interactions with polysaccharides, lipids, proteins, and other organelles of the cell (Wang et al., 2017; Khan et al., 2020b; Sher et al., 2021). At a pH of 7, the oxidation of water at the hole side results in a potential for the generation of ROS that is 1.11–1.9 eV compared to the standard hydrogen electrode [12]. As a result, the bandgap of nanomaterials is an essential component that plays a role in the formation of ROS *via* photocatalysis (Liu et al., 2016; Khan et al., 2019a, Khan et al., 2019b). Moreover, while working as photocatalysts, numerous nanomaterials produce anticancer phenomena *via* the production of ROS (Hariharan et al., 2020). Notably, photocatalysis is essential and has excellent potential in killing cancer cells effectively (Zhao et al., 2021). The development of anticancer nanomaterials with specific properties, such as low toxicity, high anticancer activity, and photo-reactivity, remains a formidable challenge.

Titanium dioxide (TiO_2_), a conventional semiconductor material, is a good choice for anticancer applications owing to its low toxicity, excellent photostability, cheap cost and strong photocatalytic performance (Tada et al., 2009; Schneider et al., 2014; Zhu et al., 2018). UV light would cause the formation of electron-hole pairs when TiO_2_ was utilized as a photocatalyst. The electron-hole pairs may combine with water or oxygen to generate reactive oxygen species, such as superoxide radicals (O_2_^•–^) and hydroxyl radicals (^•^OH) (Liu et al., 2010). These highly reactive species induce phototoxicity and cause cancer cell death. Despite TiO_2_’s photocatalytic potential, its rapid electron and hole recombination would limit its efficiency. To reduce this effect and improve the photocatalytic efficacy of TiO_2_, noble metal nanoparticles (NPs) such as Ag, Au, etc., might be utilized (Liu et al., 2010, 2016b).

**FIGURE 1:**
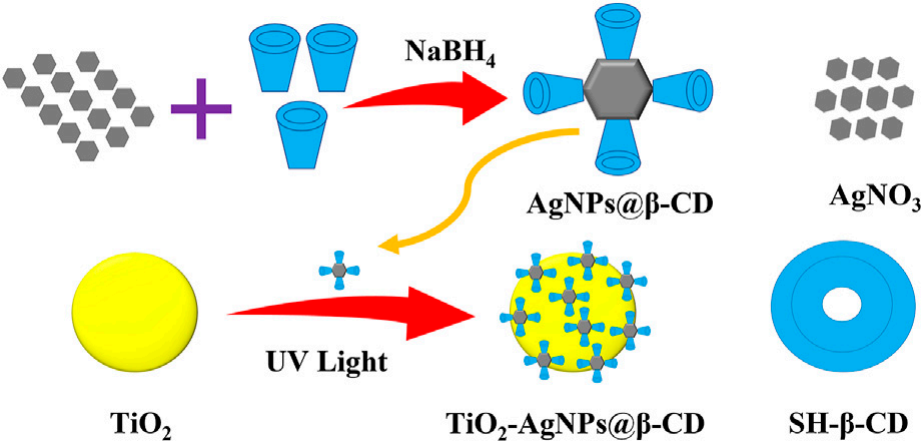
Schematic of the synthesis process of TiO_2_–Ag NPs@-CD.

To address the aforementioned grim issues, we have developed a novel photocatalyst by decorating ultrasmall silver nanoparticles (Ag NPs) with per-6-thio-β-cyclodextrin (SH-β-CD) on TiO_2_ NPs (Figure 1). Particularly, SH-β-CD can be utilized to synthesize ultrasmall Ag NPs (Ag NPs@β-CD) (Liu et al., 2000; Negishi et al., 2005), and it can also improve the interaction between Ag NPs and TiO_2_ NPs (Zhang et al., 2012; Li et al., 2015). Hence, an important element of our strategy is the utilization of SH-β-CD, which promotes the connection between Ag NPs and TiO_2_ NPs, resulting in the development of an effective photocatalyst. Moreover, through the host-guest interaction, the unique cavity in the toroidal structure of SH-β-CD makes the silver core further reachable to the organic moieties of the cells (Zhang et al., 2010; Chalasani and Vasudevan, 2013; Devi and Mandal, 2013; Yang et al., 2014; Alsbaiee et al., 2016; Wang et al., 2016). The combination of all three components— SH-β-CD ligands, a silver core, and TiO_2_ nanoparticles—produced a synergistic anticancer effect that resulted in a significant enhancement of photocatalytic-induced cytotoxicity of the material.

## Materials and methods

### Chemicals

Chemicals were commercially available and used as received. NaOH ≥99% were acquired from Merck. AgNO_3_ ≥99.0%, NaBH_4_ ≥98%, TiO_2_, Calcein-AM, and propidium iodide were purchased from Sigma-Aldrich. SH-β-CD was purchased from Shandong Binzhou Zhiyuan Bio-Technology CO., Ltd. (China). HeLa, hMSCs, and 3T3 cell lines were bought from ATCC (Manassas, USA). CellROX™ Green and dialysis tube (MWCO 6000–8,000 Da) were purchased from Thermofisher.

### Synthesis of Ag NPs@-CD

The Ag NPs@-CD was synthesized following the method reported previously with slight modifications (Zhu et al., 2018). In detail, 10 mM SH-β-CD and 40 mM AgNO_3_ solutions were made using 0.1 M NaOH and ultrapure water, respectively. For the development of Ag NPs@β-CD, 250 µL of AgNO_3_ and 286 µL of SH-β-CD were mixed in 9.2 ml of ultrapure water in a glass bottle. Then, 40 µL of a 1 M solution of NaOH was included in the reaction mixture and then mixed for 2 hours at 25°C. Then, 8.6 mg of pure NaBH_4_ was added to 2 ml of 0.1 M NaOH solution to prepare the NaBH_4_ solution. Then, 200 µL NaBH_4_ was poured into the reaction mixture and mixed for 6 hours at 25°C to obtain Ag NPs@β-CD. Then by utilizing a dialysis tube (MWCO 6000–8,000 Da), the synthesized Ag NPs@β-CD were purified by dialysis for 24 h.

### Synthesis of TiO_2_–Ag NPs@-CD

The TiO_2_–Ag NPs@-CD was synthesized following the method reported previously with slight modifications (Zhu et al., 2018) (Figure 1). 10 ml of Ag NPs@β-CD solution at 0.4 mM concentration was mixed with 10 mg of TiO_2_ powder followed by 40 min of stirring under UV irradiation. The resultant materials were then centrifuged and rinsed three times with pure water to get TiO_2_–Ag NPs@β-CD.

### Characterization

Powder X-ray diffraction spectroscopy (XRD) was utilized to determine the phase purity and crystalline nature of the TiO_2_–Ag NPs@-CD. For this purpose, Bruker D_2_ PHASER with LYNXEYE XE-T detector (Haidian, Beijing, China) was used at a wavelength (λ) of 0.154 nm. The XRD spectra were acquired in the 2θ range of 5°–60°. An energy-dispersive X-ray (EDX) spectroscopy equipment (Thermo Fisher Scientific Ultradry (Madison, WI, USA) linked to a scanning electron microscope was used to analyze the chemical composition of the generated TiO_2_–Ag NPs@-CD. A Tecnai F12 microscope (FEI/Philips Tecnai 12 BioTWIN, Baltimore, MD, USA) was utilized to obtain TEM images of the TiO_2_–Ag NPs@-CD at 200 kV acceleration voltage. Before being placed on a carbon-coated copper grid for TEM examination, the samples were mixed in methanol. Then the mixture was sonicated at 25–30°C. The copper grid was dried 5–10 min after draining the excess solution.

### Cell line and culture

HeLa, hMSCs, and 3T3 cell lines were bought from ATCC (Manassas, USA). Cells were grown in DMEM supplemented with 10% FBS (v/v), 100 U/mL penicillin, and 100 μg/ml streptomycin at 37°C in a humidified incubator with 5% CO_2_. Cells were subcultured once their confluency reached 80 percent. For tests, cells in the logarithmic growth phase were utilized.

### Cell viability analysis

MTT assay was used to analyze cell viability with slight modifications (Khan et al., 2020a; Xu et al., 2020). Briefly, HeLa cells were incubated for 2 h at 37°C in 96-well plates with 100 µL of samples at different concentrations of 0.5, 1.0, 2.0, 4.0, 8.0, 16, 32, and 64 μg/ml. Then they were irradiated with a GGZ-300W high-pressure Hg lamp (E_max_ = 365 nm) at room temperature. A UV pass filter was used to obtain a light wavelength between 300 and 400 nm. The light intensity at the liquid surface was measured by a VLX-3W radiometer-photometer (USA). The incident light intensity was 3.7 mW/cm^2^ (Zhang and Sun, 2004). Control was preprepared without treatment. Each well received 10 µL of MTT (5 mg/ml) and was incubated for 4 h. To dissolve the crystal formazan dye, 150 µL of DMSO was added to the medium, and optical density was measured at 540 nm using a Microplate Reader. The cell viability was calculated utilizing the following formula.

Cell viability (%) = (OD_s_/OD_c_) × 100

Where OD_s_ and OD_c_ are optical densities of sample and control, respectively.

### Live-dead staining assay

A live-dead staining assay was conducted following the protocol reported previously in (Xu et al., 2020). In detail, after 24 h of the incubation of HeLa cells with 100 µL of the TiO_2_–Ag NPs@-CD at 64 μg/ml, the cells were rinsed with PBS and stained with a Live-dead cell viability kit by following the manufacturer’s instructions to determine the cell viability. In summary, cells were treated with 2 and 4.5 μM of calcein-AM and propidium iodide (PI) staining solution, respectively. Following this, cells were incubated for 30 min at 37°C. The Live-dead kit determines cell viability based on the integrity of the cell membrane. CLSM (confocal laser scanning microscope) was used to observe live and dead cells, with excitation wavelengths of 490 and 535 nm for Calcein-AM and PI and emission wavelengths of 515 and 617 nm for Calcein-AM and PI, respectively. We only investigated TiO_2_–Ag NPs@-CD for the Live-dead staining experiment because they showed good cytotoxic effects on HeLa cancer cells.

### ROS and oxidative stress measurement

As previously reported (Lu et al., 2021), CellROX™ Green (C10444, Thermofisher) was used to investigate the death of HeLa cancer cells due to ROS generation. HeLa cancer cells were treated with 100 µL of TiO_2_–Ag NPs@-CD at a concentration of 64 μg/ml and incubated for 24 h at 37°C. The HeLa cancer cells were subsequently treated for an additional 30 min at 37°C with CellROX™ Green (5 µM). CLSM was then utilized to obtain pictures with excitation wavelengths of 485 nm and emission wavelengths of 520 nm. ROS generation in TiO_2_–Ag NPs@-CD treated cells was compared to untreated cells (negative control) and those treated with 1 mM H2O2 (positive control).

### Biocompatibility analysis

The MTT technique was employed to evaluate the biocompatibility of Ag NPs@-CD and TiO_2_-Ag NPs@-CD with hMSCs and 3T3 cells in terms of cell viability (%). The same protocol is repeated as described in *Cell line and culture* Section and *Cell viability analysis* Section. The 100 µL of both samples at the concentration of 64 μg/ml were used as a treatment for both cell lines.

### Statistics analysis

All biological tests were conducted in triplicate, and data are reported as the mean ± standard deviation. In addition, we utilized one-way and two-way ANOVA to determine the significant level of 0.05.

## Results and discussion

### Characterization

**FIGURE 2:**
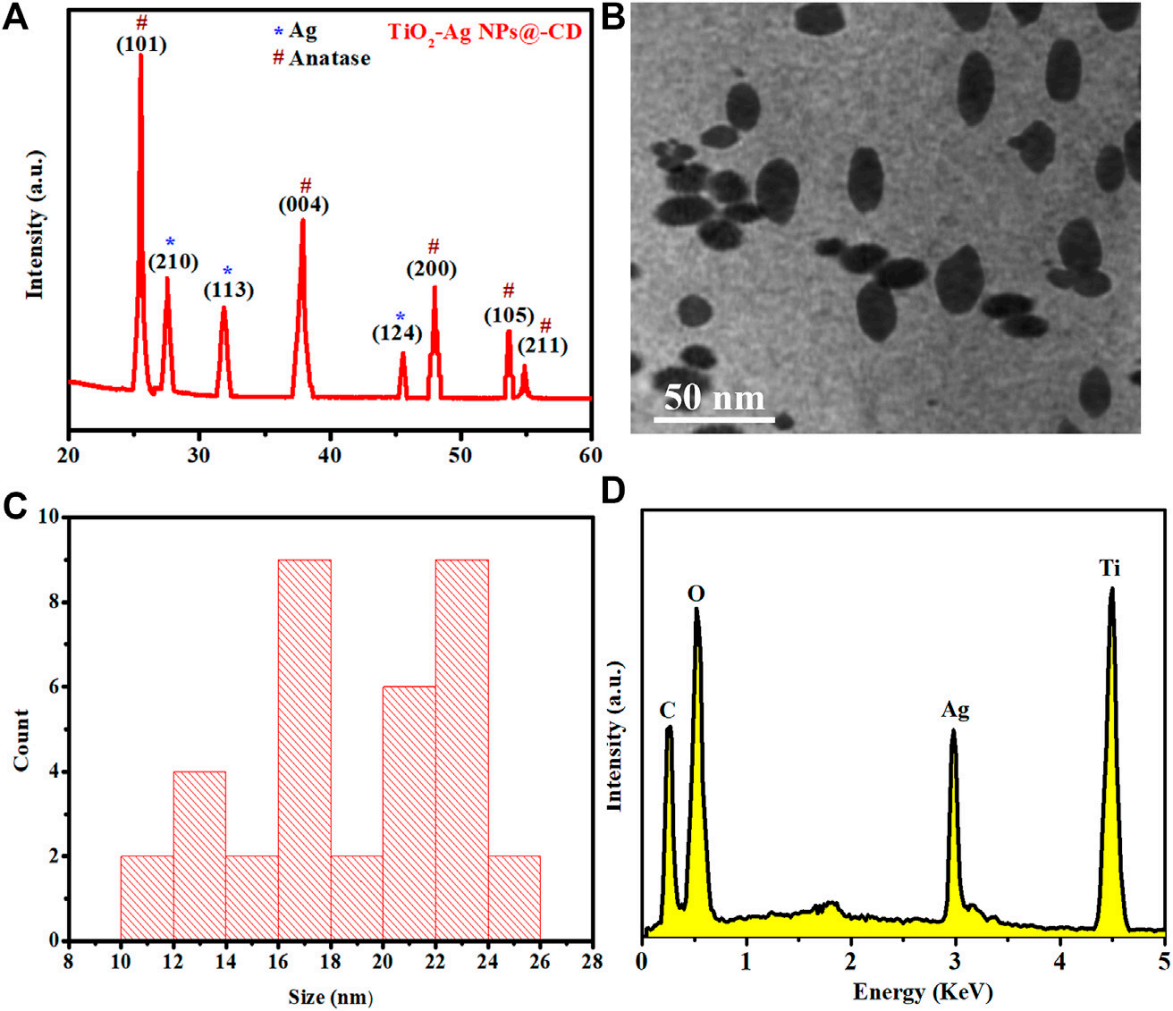
**(A)** XRD, **(B)** TEM, **(C)** size distribution, and **(D)** EDX of synthesized TiO_2_–Ag NPs@-CD.

The TiO_2_–Ag NPs@-CD were characterized for their crystallinity using XRD. Figure 2A shows the XRD spectra. XRD pattern reveals the peaks indexed to (101), (004), (200), (105), and (211) planes of anatase TiO_2_ at 2θ angles of 25.50°, 37.92°, 48.01°, 53.71°, and 54.88°, respectively. The XRD pattern of TiO_2_ is closely matched with JCPDS 21–1272 (Abdelsalam et al., 2020). No peaks associated with the rutile phase of TiO_2_ were observed. In addition, the XRD pattern also demonstrated the existence of other peaks indexed to (210), (113), and (214) crystal planes of Ag at 2θ angles of 27.51°, 31.87°, and 45.57°, respectively (Karthik et al., 2014). The peak’s intensity and their sharpness indicate that the TiO_2_–Ag NPs@-CD are highly crystalline in nature. Figure 2B shows the TEM image, which demonstrates that the TiO_2_–Ag NPs@-CD have spherical and oval morphology with uniform dispersion. No agglomeration was observed. Figure 2C shows the histogram for the size distribution determined from TEM. The histogram shows that they have an average size of 18.98 ± 4.1. The compositional analysis of TiO_2_–Ag NPs@-CD was performed using EDX. Figure 2D depicts the EDX spectrum. EDX pattern exhibits that the synthesized material is mainly composed of silver, titanium, and oxygen. Moreover, carbon peak is also evident in the EDX spectrum, which can be attributed to the CD. Abdelsalam et al. also reported similar EDX pattern results for Ag-doped TiO_2_ NPs (Abdelsalam et al., 2020).

### Cell viability analysis

**FIGURE 3:**
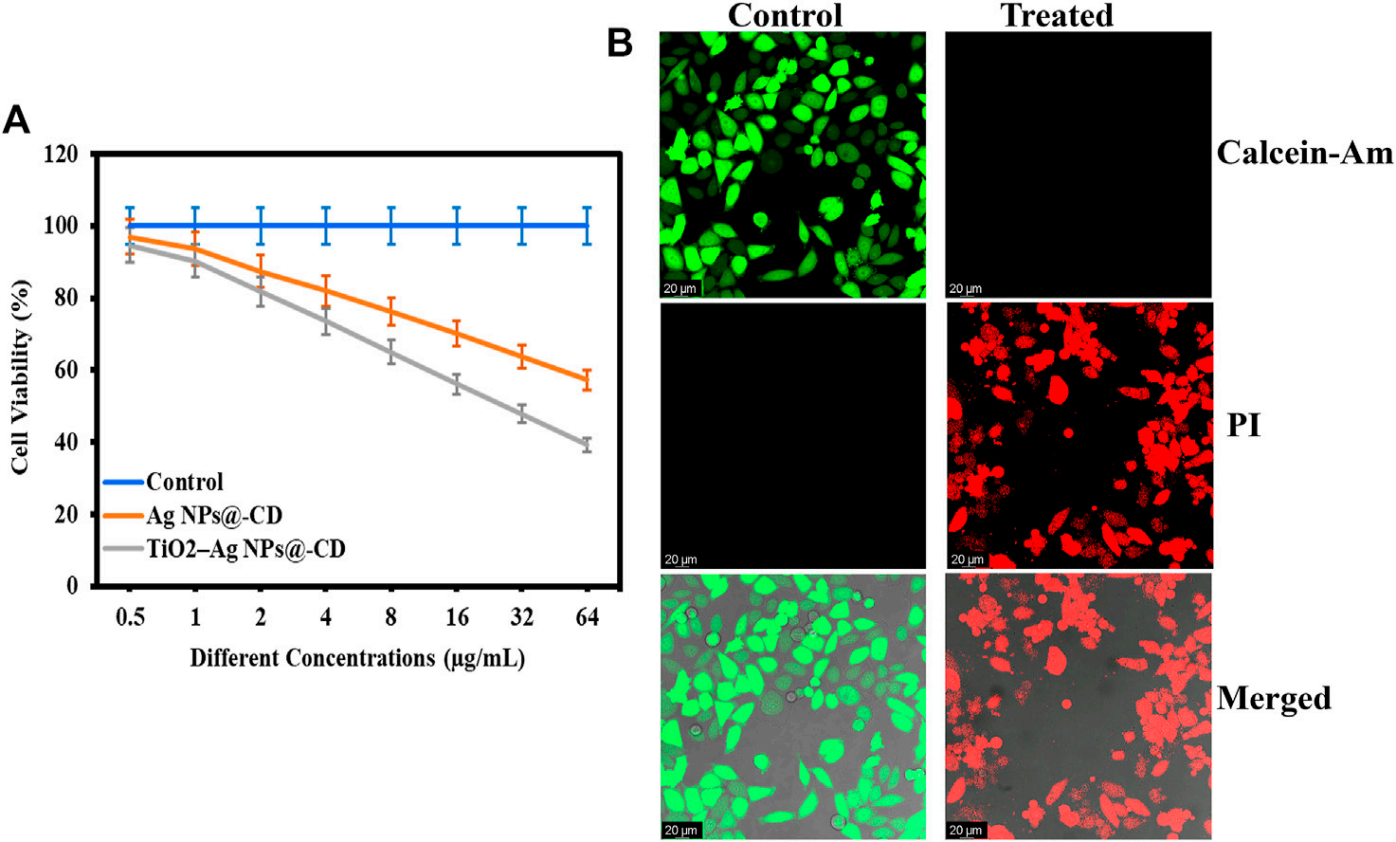
**(A)** Anticancer activity in terms of cell viability (%) against HeLa cancer cells. **(B)** Live/dead CLSM images of control and treated HeLa cancer cells with TiO_2_–Ag NPs@-CD at 64 μg/ml concentration. Data are expressed as mean ± SD; ns *p* > 0.05, **p < 0.01, ***p < 0.001.

The anticancer activity of Ag NPs@-CD and TiO_2_–Ag NPs@-CD against HeLa cancer cells was determined using an MTT assay. Figure 3A shows the results in terms of cell viability (%). Both samples presented concentration-dependent anticancer activity against HeLa cancer cells. The results further show that the least cell viability (%) of HeLa cells was manifested with TiO_2_–Ag NPs@-CD compared to Ag NPs@-CD at all tested concentrations.

### Live/dead staining analysis

A Live/dead staining assay was further performed after treatment of HeLa cancer cells with TiO_2_–Ag NPs@-CD at 64 μg/ml to affirm the inhibition of cell proliferation. The live cells were stained with Calcein-Am (Green), and the dead were labeled with PI (Red). The results are presented in Figure 3B. In contrast, to control, HeLa cells treated with TiO_2_–Ag NPs@-CD emitted stronger red fluorescence, indicating that their cell membrane had been damaged. Furthermore, treated HeLa cells displayed abnormal shape and aggregation, indicating the presence of a more significant number of apoptotic cells. As a result of these observations, it is conceivable that the anticancer activity of TiO_2_-Ag NPs@-CD is attributable to their ability to damage the cell membrane of HeLa cancer cells.

### ROS and oxidative stress analysis

**FIGURE 4:**
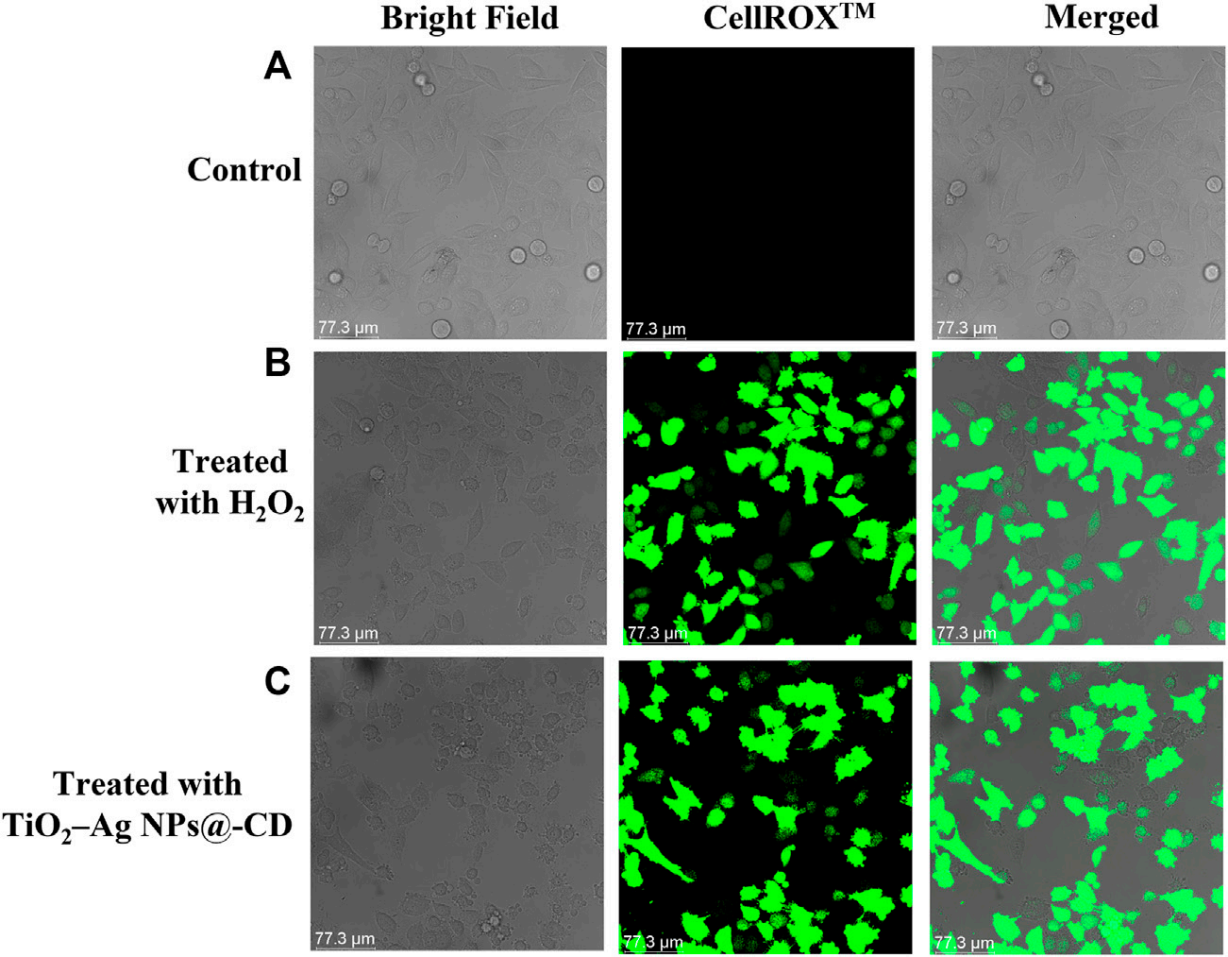
CLSM images of ROS-induced oxidative stress in **(A)** untreated (control), **(B)** treated HeLa cells with H_2_O_2_, and **(C)** TiO_2_-Ag NPs@-CD.

We have further investigated the performance of ROS-induced oxidative stress in destroying HeLa cancer cells. It is well established that nanomaterials induced apoptotic cell death in neoplastic cells *via* the oxidative stress triggered by the generation of ROS. ROS generate oxidative stress by their intercalation with different organelles inside the cells. Therefore, ROS-induced oxidative stress was by employing a CellROX™ Green staining kit. The HeLa cancer cells were treated with 100 µL of TiO_2_–Ag NPs@-CD (64 μg/ml concentration) and H_2_O_2_ (positive control) and further stained with CellROX™ Green. After incubation, the images were acquired using CLSM. As shown in Figures 4A–C, CLSM images demonstrate that untreated HeLa carcinoma cells do not produce intracellular ROS. However, HeLa cancer cells treated with TiO_2_–Ag NPs@-CD and H_2_O_2_ exhibited adequate and significant levels of green fluorescence. A similar observation was also reported by Hariharan et al. (Hariharan et al., 2020). These results imply that the ROS-induced oxidative stress caused by TiO_2_-Ag NPs@-CD within HeLa cancer cells is also a contributing factor to their extraordinary anti-cancer performance.

### Anticancer mechanism

**FIGURE 5:**
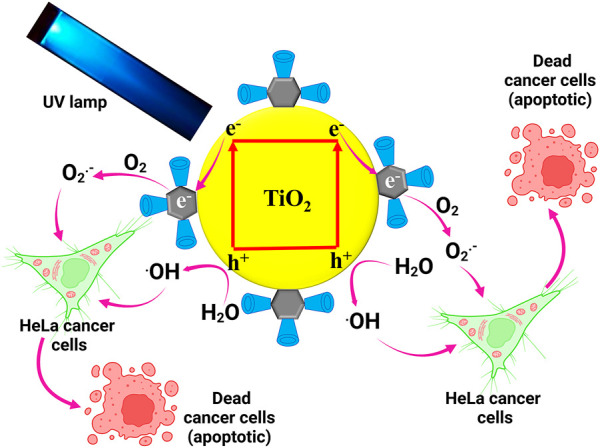
Proposed photocatalytic irradiated anticancer mechanism of synthesized TiO_2_–Ag NPs@-CD against HeLa cancer cells.

Based on the results from the experiments, we proposed the photocatalytic irradiated anticancer mechanism of synthesized TiO_2_-Ag NPs@-CD against HeLa cancer cells, as shown in Figure 5. When exposed to UV light, the excited electrons move to the Ag core, forming a new redox center. These electrons then combine with oxygen at the Ag core, producing reactive O_2_^•–^. Furthermore, the presence of SH—CD on the Ag core’s surface may efficiently trap cancer cells through a host-guest interaction. Cancer cells begin to perish when they diffuse to active locations due to the reactive O_2_^•–^. The holes, on the other hand, may react with water to produce ^•^OH radicals, which would also increase the induction of toxicity in HeLa cancer cells, leading to apoptosis. Taken together, the improved photocatalytic irradiated anticancer activity of the TiO_2_-Ag NPs@-CD may be attributed to the synergy between all three components, namely the Ag core SH—CD ligands and TiO_2_ NPs. A similar enhanced photocatalytic performance was also reported by (Zhu et al., 2018).

### Biocompatibility investigations

**FIGURE 6:**
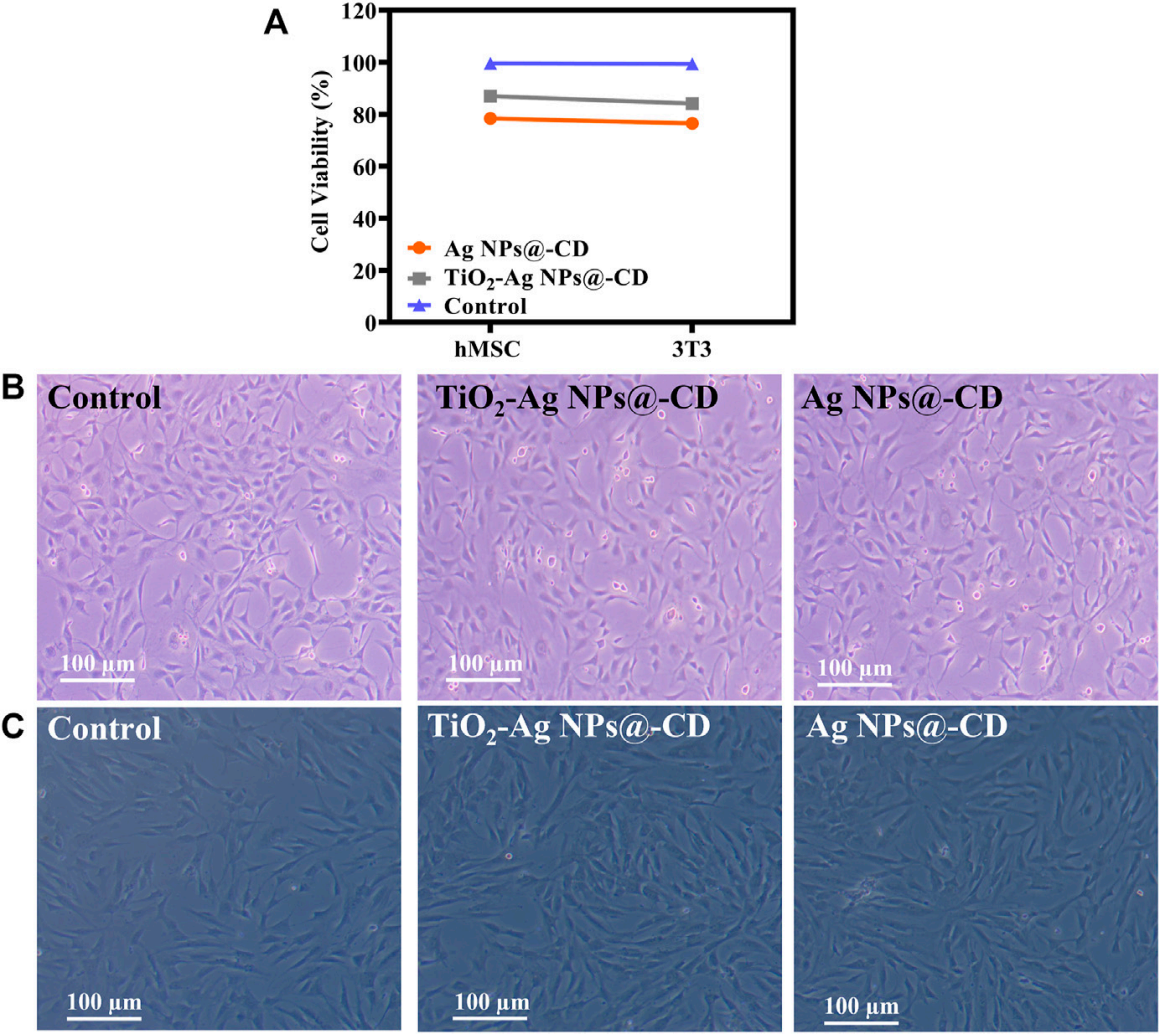
**(A)** Biocompatibility of Ag NPs@-CD and TiO_2_-Ag NPs@-CD with hMSC and 3T3 cells. Inverted micrographs of **(B)** 3T3 and **(C)** hMSC. Data are expressed as mean ± SD; ***p < 0.001, ***p < 0.001.

Biocompatibility of Ag NPs@-CD and TiO_2_-Ag NPs@-CD with hMSC and 3T3 cells was determined in terms of cell viability (%). Figure 6A shows the cell viability (%) results. The findings demonstrate that TiO_2_-Ag NPs@-CD exhibited high biocompatibility with hMSC and 3T3 cells. However, it seems that hMSC cells are more biocompatible. While Ag NPs@-CD exhibited lower biocompatibility with hMSC and 3T3 cells than TiO_2_-Ag NPs@-CD.

Using an inverted microscope, we next examined the morphologic vicissitudes in hMSC and 3T3 cells treated with TiO_2_-Ag NPs@-CD and Ag NPs@-CD at a concentration of 64 μg/ml. Figures 6B,C illustrate the inverted micrographs of 3T3 and hMSC cells, respectively. After treatment with TiO_2_-Ag NPs@-CD, the morphology of hMSC and 3T3 cells was comparable to that of the control group (untreated cells). However, Ag NPs@-CD was marginally toxic to hMSC and 3T3 cells as there was a slight change in the shape and size of the cells. The findings of inverted microscopy and cell viability were found to be comparable. As a result, the improved biocompatibility may be attributed to TiO_2_ NPs contained in TiO_2_-Ag NPs@-CD.

## Conclusion

In summary, we successfully synthesized novel TiO_2_-Ag NPs@-CD and explored their anticancer effect on HeLa cancer cells *in vitro*. We have determined that cell membrane disintegration and ROS-induced oxidative stress generated by TiO_2_-Ag NPs@-CD inside HeLa cancer cells are the contributing factors to their exceptional anti-cancer performance. TiO_2_-Ag NPs@-CD is highly biocompatible with both hMSC and 3T3 cells, which indicates their importance in being employed in pharmacological and clinical applications. Future research is required to assess the dose-dependent *in vivo* cytotoxic and biocompatibility. In addition, this work will provide the opportunity for the continued development of biocompatible materials with improved biological properties.

## Data Availability

The raw data supporting the conclusions of this article will be made available by the authors, without undue reservation.
